# Cost-effectiveness of PARP inhibitors in malignancies: A systematic review

**DOI:** 10.1371/journal.pone.0279286

**Published:** 2022-12-15

**Authors:** Haiying Ding, Chaoneng He, Yinghui Tong, Qilu Fang, Xiufang Mi, Lingya Chen, Wenxiu Xin, Luo Fang

**Affiliations:** Department of Pharmacy, Zhejiang Cancer Hospital, Institute of Basic Medicine and Cancer (IBMC), Chinese Academy of Sciences, Hangzhou, China; University of Salemo, ITALY

## Abstract

**Objectives:**

Poly (ADP-ribose) polymerase inhibitor (PARPi) have become a mainstay for the treatment of BRCA-mutant malignancies. PARPis are likely to be more effective but also bring an increase in costs. Thus, we aimed at evaluating the cost effectiveness of PARPis in the treatment of malignancies.

**Methods:**

Studies of cost effectiveness of PARPis were searched from PubMed, Web of Science, and Cochrane Library. Key information was extracted from the identified studies and reviewed. Quality of the included studies was evaluated using Quality of Health Economic Studies (QHES) instrument. Modeling techniques, measurement of parameters and uncertainty analysis were analyzed across studies. Interventions and cost-effectiveness results were reported stratified by patient population.

**Results:**

Among the 25 studies identified, we included 17 on ovarian cancer, 2 on breast cancer, 3 on pancreatic cancer, and 3 on prostate cancer that involved olaparib, niraparib, rucaparib, and talazoparib. All studies had a QHES score of above 75. In the maintenance therapy of ovarian cancer, additional administration of olaparib was cost-effective for newly diagnosed patients after first-line platinum-based chemotherapy but was not cost-effective for platinum-sensitive recurrent patients in majority studies. However, the economic value of other PARPis in ovarian cancer as well as all PARPis in other tumors remained controversial. Cost-effectiveness of PARPi was primarily impacted by the costs of PARPi, survival time, health utility and discount rate. Moreover, genetic testing improved the cost-effectiveness of PARPi treatment.

**Conclusions:**

PARPi is potentially cost-effective for patients with ovarian, pancreatic, or prostate cancer. Genetic testing can improve the cost-effectiveness of PARPi.

## Introduction

Poly ADP-ribose polymerase (PARP) is a DNA repair enzyme. PARP1 and PARP2 are involved in the DNA damage response, cell transcription, apoptosis, and immune function [1–3]. PARP inhibitor (PARPi) inhibits the recruitment of DNA repair protein by capturing PARP1 and PARP2 on DNA damage sites, blocking the mitotic catastrophe of tumor cells. Further, PARPi selectively promotes the apoptosis of tumor cells that have homologous recombination deficiency (HRD), such as the BRCA-mutated tumor cells [4].

PARPis (olaparib, niraparib, rucaparib, and talazoparib), have recently been approved by the United States (US) Food and Drug Administration and have been used to treat ovarian, breast, prostate, and pancreatic cancers, thereby improving the survival of cancer patients and reduce the risk of disease progression or death [5–12].

Owing to the high cost of PARPis, their economic evaluation is gaining attention. Gao W et al. [13] reviewed the cost-effectiveness of PARPi in the treatment of advanced ovarian cancer published before June 2019, focusing on the methodology reliability and factors affecting the economy. In addition to ovarian cancer, cost-effectiveness studies of PARPi for breast cancer, pancreatic cancer, and prostate cancer have also been published. However, to the best of our knowledge, no study has summarized the cost-effectiveness of PARPi in the treatment of these various tumors. Moreover, after 2020, a number of economic studies on the treatment of ovarian cancer with PARPi have been published, and data need to be updated. Therefore, we reviewed the literature on the cost-effectiveness of PARPi in treatment of malignancies and aimed at providing guidance for clinical decision making.

## Methods

### Literature search

The study protocol was registered with PROSPERO, and the study was performed following the Preferred Reporting Items for Systematic Reviews and Meta-Analyses (PRISMA) guidelines (S1 File) [14]. We searched PubMed, Web of Science, and Cochrane Library for studies related to the economic evaluation of PARPi published between January 1, 1998, to September 30, 2022. The main search terms included PARP, Poly (ADP-ribose) Polymerase Inhibitors, olaparib, niraparib, rucaparib, talazoparib, veliparib, fluzoparib, pamiparib, cost, economic. The search strategies are detailed in the S2 File.

### Eligibility criteria

Studies fulfilling the following criteria were included in review: (1) patients with malignancies were the target population; (2) PARPi was used as the intervention strategy; (3) the study design involved a cost-effectiveness analysis; (4) pharmacoeconomic outcomes were reported, including cost, health outcomes (life-years [LY], quality-adjusted life-years [QALYs]), and incremental cost-effectiveness ratio (ICER). Studies were excluded if they were (1) not written in English; (2) conference abstracts, editorials, literature reviews, case reports, comments, notes, or letters; (3) unavailable for full manuscript.

### Data extraction and processing

All literature screening and data extraction were independently performed by two researchers (HD and CH). Disagreements were discussed and resolved with a third researcher (WX). The extracted content primarily included first author, country, publication journal and year, evaluation type, cancer type, study population, research perspective, time horizon, source of cost and effectiveness, modeling method, and findings, etc.

### Literature quality evaluation

The Quality of Health Economic Studies (QHES) instrument was used to evaluate the quality of all studies included [15]. It is a validated instrument designed to assess the appropriateness of the research methodology as well as the validity, transparency, and comprehensiveness of research findings through 16 items. A QHES score of 0–24 indicates very poor quality, 25–49 indicates low quality, 50–74 indicates medium quality, and 75–100 indicates high quality [15]. The QHES evaluation was performed independently performed by two researchers.

## Results

### Literature search results

**Fig 1:**
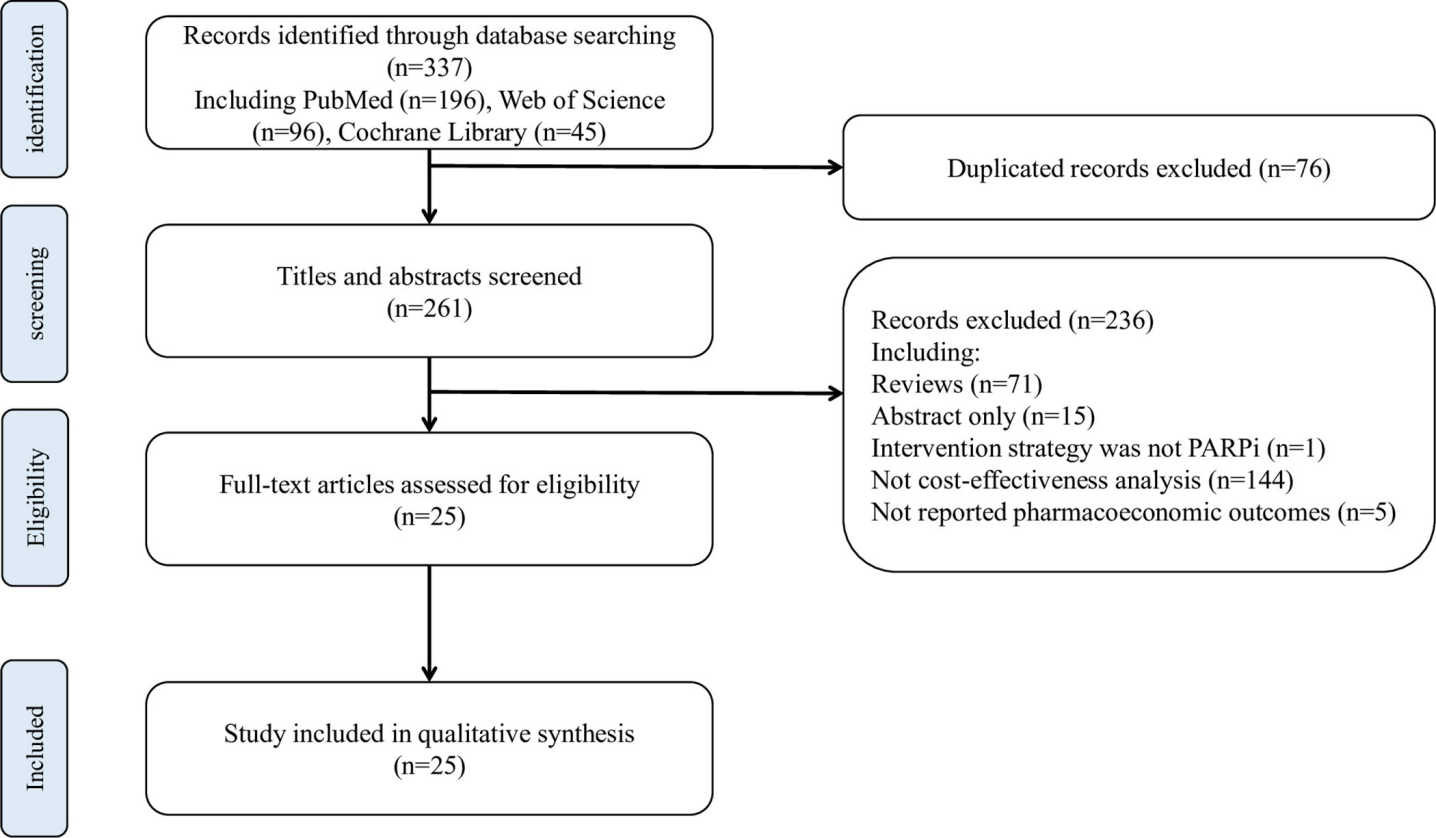
Flowchart of the study identification and selection process.

We retrieved 337 studies, among which 76 were duplicated. After titles and abstract screening, 236 studies were excluded, and 25 studies were finally included [16–40]. The flow diagram of the study search and selection process was shown in Fig 1.

### Quality evaluation results

The results of the QHES quality assessment are presented in S1 Table. All included studies are classified as high quality. Their objectives, perspective of the analysis, measurement of costs and health outcomes were all clearly elaborated. The most common issues were the absence of detailed description for time horizon or discounting [17, 19, 23, 26, 28, 33] as well as lack of research funding disclosure [16–18, 28, 34]. In addition, one study did not conduct the uncertainty analysis [28], and two did not describe detailed information of the model structure [17, 23].

### Characteristics of the included studies

Characteristics of the included studies are shown in presented in S2 Table. Overall, 19 studies were published after June, 2019, including 9 in 2020 [22–30], 7 in 2021 [31–37], and 3 2022 [38–40]. Studies were conducted from the United States (US) (n = 13) [16–20, 23–28, 38, 40], China (n = 5) [27, 30, 32, 39, 40], Singapore (n = 2) [31, 34], Japan [21], Italy [22], and Spain [33]. PARPi evaluated in these studies included olaparib [16–18, 20–22, 25–28, 30–32, 34–40], niraparib [18–20, 23, 26, 28], rucaparib [20, 28], talazoparib [33], and veliparib [24]. Modeling methods employed included the Markov model (n = 11) [16, 21, 22, 24, 28, 30, 32, 36, 37, 39, 40], decision tree model (n = 6) [17–20, 23, 35], partitional survival model (n = 7) [25, 27, 31, 33, 34, 38], and the non-Markov alternatives (n = 1) [26].

The clinical data in the included studies were primarily derived from randomized controlled trials (RCT). Clinical outcomes included progression-free survival (PFS), overall survival (OS), and adverse drug reaction. The models were either based on a single trial (n = 19) or multiple clinical trials (n = 6) [18, 20, 24, 26, 28, 35]. Most studies were assessed from the perspective of health care system (n = 10) [18, 22, 26, 31–33, 35, 38–40], third-party payers (n = 10) [17, 20, 21, 23–25, 27, 28, 34, 36] and the whole society (n = 3) [16, 19, 30]. Most studies reported a 3% discount rate on the cost and effects, and two studies reported a discount rate of 5% [32, 40] and one reported 2% [21].

Incremental cost-effectiveness analyses were included in all studies. In addition, the quality-adjusted life year (QALY), progression free-QALY [19], and progression-free life year (PF-LY) [18, 26, 35] were also used as parameters to measure the effectiveness. Apart from Wolford et al. [28], all other authors performed uncertainty analysis. Among them, authors from 13 studies conducted one-way sensitivity analysis (OWSA) and probabilistic sensitivity analysis (PSA) [16, 20–22, 26, 27, 30, 32, 34, 37, 39, 40], those from six studies conducted OWSA, PSA and scenario analysis (SA) [18, 25, 31, 35, 36, 38], those from one study conducted OWSA, two-way sensitivity analysis (TWSA) and PSA [24], those from three studies only conducted OWSA (n = 3) [19, 23, 33], and those from one only performed SA [17].

### Cost-effectiveness outcomes

Cost-effectiveness outcomes are summarized in Table 1 and S3 Table. Among 17 studies on the cost-effectiveness of PARPi maintenance therapy for ovarian cancer, eight were for platinum-sensitive recurrent ovarian cancer [16–20, 31, 35, 39] and nine were for first-line platinum-based chemotherapy for newly diagnosed ovarian cancer [22–26, 28, 34, 37, 38].

**Table 1:** Overview of the cost-effectiveness outcomes.

Interventions	Results	Groups	Perspective	WTP threshold	References
**Maintenance therapy for platinum-sensitive recurrent ovarian cancer**					
olaparib vs. no maintenance	cost-effective	with gBRCA mutation non-gBRCA mutation	US payer	$150,000/QALY	Guy et al. (2019) [20]
		all patients with gBRCA mutation	Taiwan (China) single-payer	$93,478/PF-LYS	Leung et al. (2021) [35]
	not cost-effective	non-gBRCA mutation			
		with gBRCA mutation	US societal	$50,000-$100,000/ PF-LYS	Secord et al. (2013) [16]
		with gBRCA mutation wild-type gBRCA	US third-party payer	$50,000-$100,000/PF-LYS	Smith et al. (2015) [17]
		all patients with gBRCA mutation non-gBRCA mutation	US health care sector	$100,000/PF- LYS	Zhong et al. (2018) [18]
		all patients with gBRCA mutation	Singapore healthcare system	$34,047/QALY	Cheng et al. (2021) [31]
		with gBRCA mutation	Chinese healthcare system	$31,498.70/QALY	Shu et al. (2022) [39]
niraparib vs. no maintenance	cost-effective	with gBRCA mutation non-gBRCA mutation	US payer	$150,000/QALY	Guy et al. (2019) [20]
		all patients with gBRCA mutation	Taiwan (China) single-payer	$93,478/PF-LYS	Leung et al. (2021) [35]
	not cost-effective	non-gBRCA mutation			
		all patients with gBRCA mutation non-gBRCA mutation	US health care sector	$100,000/PF-LYS	Zhong et al. (2018) [18]
		with gBRCA mutation with gBRCA mutation or HRD	US societal	$100,000/PF-QALY	Dottino et al. (2019) [19]
rucaparib vs. no maitenance	not cost-effective	with gBRCA mutation non-gBRCA mutation	US payer	$150,000/QALY	Guy et al. (2019) [20]
olaparib vs. niraparib	cost-effective	all patients with gBRCA mutation non-gBRCA mutation	Taiwan (China) single-payer	$93,478/PF-LYS	Leung et al. (2021) [35]
olaparib vs. niraparib vs. rucaparib	Niraparib is most cost-effective, rucaparib is most non-cost-effective	with gBRCA mutation non-gBRCA mutation	US payer	$150,000/QALY	Guy et al. (2019) [20]
**Maintenance therapy after first-line platinum-based chemotherapy for newly diagnosed ovarian cancer**					
olaparib vs. no maintenance	cost-effective	with gBRCA mutation	Italian NHS	€16,372/QALY	Armeni et al. (2020) [22]
		with gBRCA mutation	US third-party payer	$100,000/QALY	Muston et al. (2020) [25]
		with gBRCA mutation	Singapore healthcare payer	$43,799/QALY	Tan et al. (2021) [34]
		with gBRCA mutation	Spanish NHS	€25,000/QALY	Moya-Alarcón et al. (2021) [37]
	not cost-effective	with gBRCA mutation HRD without BRCA mutation with HRP	US health care sector	$100,000/PF-LYS	Penn et al. (2020) [26]
niraparib vs. no maintenance	cost-effective	all patients HRD group HRD without BRCA mutation	US third-party payer	$100,000/QALY	Barrington et al. (2020) [23]
	not cost-effective	with gBRCA mutation HRD without BRCA mutation with HRP	US health care sector	$100,000/PF-LYS	Penn et al. (2020) [26]
olaparib+bevacizumab vs. observation	not cost-effective	with gBRCA mutation HRD without BRCA mutation with HRP	US health care sector	$100,000/PF-LYS	Penn et al. (2020) [26]
olaparib+bevacizumab vs. bevacizumab	cost-effective	HRD group	US healthcare system	$100,000/QALY	Elsea et al. (2022) [38]
**Breast cancer first-line therapy**					
olaparib vs. standard chemotherapy	not cost-effective	with gBRCA mutation	Japanese healthcare payer	$107,143/QALY	Saito et al. (2019) [21]
talazoparib vs. standard chemotherapy	not cost-effective	with gBRCA mutation	Spanish NHS	€21,000, €24,000, €25,000, €60,000 /QALY	Olry de Labry Lima et al. (2021) [33]
**Pancreatic cancer maintenance therapy**					
olaparib vs. no maintenance	cost-effective	with gBRCA mutation	US healthcare systems	$50,000/QALY	Li et al. (2021) [32]
			China healthcare systems	$30,829/QALY	Li et al. (2021) [32]
	not cost-effective	with gBRCA mutation	US Payer	$200,000/QALY	Wu et al. (2020) [27]
			Chinese society	$28,255.55/QALY	Zhan et al. (2020) [30]
**Metastatic castration-resistant prostate cancer second-line treatment**					
olaparib vs. standard care	cost-effective	with BRCA 1/2 or ATM mutation has alterations in any of all 15 prespecified genes	US payer	$150,000/QALY	Su et al. (2020) [27]
		with BRCA 1/2 or ATM mutation	US health service	$150,000/QALY	Xu et al (2022) [40]
	not cost-effective	with BRCA 1/2 or ATM mutation	US payer	$200,000/QALY	Li et al. (2021) [36]
			Chinese health service	¥217,341/QALY	Xu et al. (2022) [40]

WTP indicates willingness to pay; US, United States; QALY, quality adjusted life year; PF-LYS, progression-free life year saved; NHS, National Health Service; HRD, homologous recombination deficiency; HRP, homologous recombination proficiency.

For the maintenance treatment in platinum-sensitive recurrent ovarian cancer, olaparib was cost-effective from the payers’ perspective in the US [20] and Taiwan (China) [35] when compared with placebo but not cost-effective from the perspective of US society [16], third-party payer [17], health care sector [18], Singapore healthcare system [31] and Chinese healthcare system [39]. Niraparib was also cost-effective from the payers’ perspective in US [20] and Taiwan (China) [35] but not from the perspective of the US health care sector [18] and whole society [19]. Rucaparib was not cost-effective compared with routine surveillance from the perspective of US payer [20]. In addition, a comparison among PARPis revealed that olaparib was more cost-effective than niraparib from Taiwan (China) payer’s perspective [35]; however, niraparib was the most cost-effective, followed by olaparib and rucaparib, from the payer’s perspective in the United States [20].

Regarding the maintenance treatment after first-line platinum-based chemotherapy for newly diagnosed ovarian cancer, most studies revealed that olaparib was more cost-effective than routine surveillance [22, 23, 25, 34] except for one study from the US health care sector perspective that showed olaparib was not cost-effective at the WTP threshold of $100, 000/PF-LY [26]. In the United States, niraparib was cost-effective from the perspective of third-party payer [23], but not from perspective of the health care sector [26]. Elsea et al. demonstrated that olaparib plus bevacizumab is cost-effective compared with bevacizumab alone for the first-line maintenance treatment of HRD positive advanced ovarian cancer from the perspective of a US healthcare system. In addition, two study did not conclude cost-effectiveness of PARPi compared with placebo [24, 28]. Gonzalez et al. [24] compared a “PARPi-for-all” to a biomarker-directed frontline maintenance therapy approach. Wolford et al. did not set a WTP threshold and did not conclude on whether PARPi was cost-effective [28].

In the treatment of metastatic breast cancer, studies on olaparib in Japan [21] and on talazoparib in Spain [33] revealed that compared with standard chemotherapy, PARPi was not cost-effective from the perspective of a healthcare system. Compared with placebo for the maintenance therapy of pancreatic cancer with gBRCA mutation, olaparib was more cost-effective from the perspective of healthcare systems in US and China [32] but not from the perspective of the Chinese society [30]. A study from the US payer perspective revealed that, in the base case analysis, olaparib was not cost-effective than placebo in the treatment of pancreatic cancer; however, PSA indicated an approximately 54% probability of olaparib being a cost-effective strategy at the threshold of $200, 000/QALY [27]. Two studies compared the cost-effectiveness of PARPi with that of a standard treatment for metastatic castration-resistant prostate cancer (MCRPC) from the payer’s perspective in the United States [27, 36]. One study showed that genomic test-directed olaparib is a preferred option [27]; on the contrary, another study revealed that olaparib is not cost-effective in patients with specific gene mutations with an ICER of $248, 248/QALY [36]. Another study indicated that olaparib is not cost effective in treatment of patients with mCRPC in China but it is cost saving in the US from perspective of health service.

### Results of sensitivity analysis

The cost-effectiveness of PARPi was primarily affected by the costs of PARPi [16–19, 22–25, 27, 30–33, 35, 36], survival time including PFS and OS [16–18, 20, 21, 23–25, 27, 35, 36], health state utilities [20, 23, 27, 32, 36] and discount rate [25, 31, 32, 34, 37]. Cost of PARPi plays as a critical factor. If cost of PARPis were decreased, such that the cost of olaparib was decreased by 52% [16] or 63% [17] in the treatment of recurrent ovarian cancer, niraparib by 62% in the treatment of recurrent ovarian cancer patients with gBRCA mutation [19], talazoparib by 85% in the treatment of breast cancer [33], olaparib by 20% in the treatment of metastatic pancreatic cancer patients with a gBRCA mutation [30], PARPi therapy would be cost-effective. Although cost of PARPis and survival time were the most sensitive factors of ICER, when these parameters were varied over the range of possible values, the ICERs remained above [18, 26, 31, 36] or below the WTP threshold [32, 34]. Cost parameters, such as the cost of treatment for adverse events [16, 19, 23, 26, 27, 30], BRCA or HRD testing [16, 19], standard chemotherapy [21, 34], relapse chemotherapy regimens, and hospice care [16], weakly affected ICER. The incidence of BRCA1/2 mutations [17, 19, 23, 24, 31] also had a minor influence on the outcome.

Genetic testing can improve the cost-effectiveness of PARPi. In the gBRCA-only group of recurrent ovarian cancer (niraparib only used in patients with gBRCA mutation after BRCA testing) and the gBRCA- and HRD-only group (niraparib used in patients with gBRCA mutation or HRD positive after BRCA and HRD testing), ICER dropped by approximately 90% compared with that in the treat-all group [19]. Another study showed that olaparib and niraparib had lower ICERs in the group with gBRCA mutation than in the overall patient population for patients with recurrent ovarian cancer [35]. Targeted treatment guided by HRD and BRCA testing can improve the cost-effectiveness of PARPi for first-line maintenance therapy of ovarian cancer [23]. In addition, PARPis (including olaparib, niraparib and veliparib) as first-line maintenance therapy for all newly diagnosed ovarian cancer was not cost-effective compared with a biomarker-directed approach (only for patients with either gBRCA mutations or HRD positive) [24].

## Discussion

PARPi is an important treatment method for ovarian, BRCA-mutated breast, prostate, and pancreatic cancer. Its cost-effectiveness is attracting the attention of global research. Several studies focusing on PARPi for malignant tumor treatment have been published [16–28, 30–37]. Gao et al [13] systematically reviewed the cost-effectiveness studies of PARPi in advanced ovarian cancer before June 2019. In contrast with this previously published study, we performed a more comprehensive review of the cost-effectiveness studies of PARPi in the treatment of ovarian, breast, pancreatic, and prostate cancers. Moreover, all research manuscript retained in this study were high-quality full texts.

We observed that the cost-effectiveness of PARPi differs with country and region, perspective of the analysis, modeling method, and parameter settings. Two studies from the US health care sector and societal perspective revealed that the use of niraparib in the maintenance treatments of platinum-sensitive recurrent ovarian cancer was not cost-effective [18, 19]. In contrast, an evaluation from the US payers’ perspective in 2019 revealed that niraparib maintenance therapy was more cost-effective than placebo [20]. This finding was consistent with those of a similar study conducted in 2021 [35], where both olaparib and niraparib were cost-effective. Regarding the comparison between different PARPis, Guy et al. [20] demonstrated that niraparib dominated olaparib and rucaparib from the perspective of payer in the US, while a study in Taiwan (China) [35] in 2019 showed that olaparib was more cost-effective than niraparib from a single-payer perspective. These inconsistencies could be attributed to the different regions in which the studies were conducted as well as the differences in research perspectives. Furthermore, Zhong et al. [18] and Dottino et al. [19] used PF-YLS to measure the effectiveness outcome, whereas Guy et al. [20] used QALY. To date, there are no clear recommendations on the use of PF-YLS with respect to the WTP threshold. Similarly, although the three studies [27, 30, 32] evaluating the cost-effectiveness of PARPi in pancreatic cancer maintenance treatment were based on the same clinical data [10], the different perspectives of analysis led to different cost-effectiveness results. In addition, the two studies assessing the cost-effectiveness of olaparib in the treatment of MCRPC still obtained contrasting results despite having the same research perspective, owing to the use of the partition survival model and Markov model, respectively, as well as a difference in the input parameters such as utility [27, 36].

A review of the sensitivity analysis of the included studies showed that the major influence on the cost-effectiveness of PARPi maintenance treatment included factors directly influencing output (costs and QALYs) such as PARPi cost, survival time, and health state utilities. Among them, the cost of PARPi is the easiest factor to modify. Therefore, local economic evaluation results can guide decision-making such as national reimbursement drug negotiation and patient assistance program, to reduce the cost of PARPi, making it a cost-effective treatment option. In addition, targeted therapy guided by biomarker detection, such as BRCA and HRD, could also improve the cost-effectiveness of PARPi [16, 17, 19, 23, 24, 35].

We encountered some limitations in the study process. First, the included studies was all based on clinical trials. Data obtained from clinical trials with strict eligibility criteria could not comprehensively reflect real-world treatment effects, limiting the generalizability of the findings. Second, owing to significant differences in modeling methods, research perspectives, and outcomes reported among studies, a meta-analysis cannot be performed in this systematic review. Third, most of the included studies were placebo-controlled, and head-to-head clinical effect data were not available to compare the cost-effectiveness between different PARPis. Therefore, a real-world economic study is recommended to further evaluate the cost-effectiveness of PARPis.

## Conclusions

PARPi is potentially cost-effective in the treatment of patients with ovarian cancer, pancreatic cancer, or prostate cancer. Its expensive nature is the major factor affecting its cost-effectiveness. Moreover, genetic testing improves the cost-effectiveness of PARPi.

## Supporting information

S1 File PRISMA checklist. (DOCX)

S2 File Search strategy. (DOCX)

S1 Table Quality assessment results using Quality of Health Economic Studies instrument. (XLSX)

S2 Table Characteristics of the included studies. (XLSX)

S3 Table Interventions and outcomes of the included studies. (DOCX)
