# Supplementary material for: Cost-effectiveness of PARP inhibitors in malignancies: A systematic review
Source: PLoS One. 2022 Dec 15;17(12):e0279286. doi: 10.1371/journal.pone.0279286 (PMC9754183; doi:10.1371/journal.pone.0279286)
Supplement: S2 File — (DOCX) [file pone.0279286.s002.docx]

**S2 File. Search strategy**

**Pubmed (N=196)**

1. Poly(ADP-ribose) Polymerase Inhibitors[MeSH]
2. Poly(ADP-ribose) Polymerase Inhibitors[Title/Abstract]
3. PARP[Title/Abstract]
4. olaparib[Supplementary Concept]
5. olaparib[Title/Abstract]
6. rucaparib[Supplementary Concept]
7. rucaparib[Title/Abstract]
8. niraparib[Supplementary Concept]
9. niraparib[Title/Abstract]
10. talazoparib [Supplementary Concept]
11. talazoparib[Title/Abstract]
12. fluzoparib[Title/Abstract]
13. pamiparib[Supplementary Concept]
14. pamiparib[Title/Abstract]
15. #1 OR #2 OR #3 OR #4 OR #5 OR #6 OR #7 OR #8 OR #9 OR #10 OR #11 OR #12 OR #13 OR #14
16. Economics[MeSH Terms]
17. Economics[Title/Abstract]
18. cost[Title/Abstract]
19. cost*[Title/Abstract]
20. #16 OR #17 OR #18 OR #19
21. #15 AND #20

**Web of Science (N=96)**

TS = (Poly (ADP-ribose) Polymerase Inhibitors OR PARP) AND TS = (cost* OR economic*) AND TS = (olaparib OR rucaparib OR niraparib OR talazoparib OR fluzoparib OR pamiparib)

**Cochrane Library (N=45)**

#1 MeSH descriptor: [Poly (ADP-ribose) Polymerase Inhibitors] explode all trees

#2 (PARP):ti,ab,kw OR (Poly(ADP-ribose) Polymerase Inhibitors):ti,ab,kw OR (olaparib):ti,ab,kw OR (niraparib):ti,ab,kw OR (rucaparib):ti,ab,kw (Word variations have been searched)

#3 (talazoparib):ti,ab,kw OR (fluzoparib):ti,ab,kw OR (pamiparib):ti,ab,kw (Word variations have been searched)

#4 MeSH descriptor: [Economics] explode all trees

#5 (Economic):ti,ab,kw OR (cost):ti,ab,kw (Word variations have been searched)

#6 (#1 OR #2 OR #3) AND (#4 OR #5)
