# Supplementary material for: Cost-effectiveness of PARP inhibitors in malignancies: A systematic review
Source: PLoS One. 2022 Dec 15;17(12):e0279286. doi: 10.1371/journal.pone.0279286 (PMC9754183; doi:10.1371/journal.pone.0279286)
Supplement: S3 Table — (DOCX) [file pone.0279286.s005.docx]

S3 Table. Interventions and outcomes of the included studies

| **Author (year)** | **Comparator** | **Interventions** | **Cost** | **Effectiveness** | **WTP threshold** | **ICER** | **Conclusions** |
| --- | --- | --- | --- | --- | --- | --- | --- |
| **Maintenance therapy for platinum-sensitive recurrent ovarian cancer** | | | | | | | |
| Secord, A. A. et al (2013) ^(16)^ | Observation | (1) Olaparib  (2) BRCA1/2 testing; treat mutation carriers with olaparib | (1) Observation: $18,960  (2) Olaparib: $70,300  (3) Treat mutation carriers with olaparib: $30,478 | PFS (months):  (1) Observation: 6.4  (2) Global olaparib: 9.2  (3) Treat mutation carriers with olaparib: 7.1 | $50,000-$100,000/ PF-YLS | Treat mutation carriers with olaparib vs observation: $193,442/PF-YLS  Olaparib vs Treat mutation carriers with olaparib: $234,128/PF-YLS | Maintenance olaparib is not cost-effective regardless of whether BRCA1/2 testing is used to direct treatment from the US societal perspective. |
| Smith, H. J. et al  (2015) ^(17)^ | Observation | Olaparib | gBRCA1/2 mutation:  (1) Observation: $5,500,000 (2) Olaparib: $169,200,000  wild-type BRCA1/2:  (1) Observation: $22,100,000  (2) Olaparib: $444,200,000 | PFS (months):  gBRCA1/2 mutation:  (1) Observation: 4.3  (2) Olaparib: 11.2  wild-type BRCA1/2:  (1) Observation: 5.5  (2) Olaparib: 7.4 | $50,000-$100,000 /PF-YLS | gBRCA1/2 mutation:  $258,864/PF-YLS  wild-type BRCA1/2: $600,552/PF-YLS | For patients with or without gBRCA1/2 mutation, maintenance olaparib is not cost-effective from a US third-party payer perspective. |
| Zhong, L. et al (2018) ^(18)^ | Placebo | (1) Olaparib  (2) Niraparib | All patients:  (1) Placebo: $1,200  (2) Olaparib: $123,200  (3) Niraparib: $138,000  Patients with gBRCA mutations:  (1) Placebo: $1,600  (2) Olaparib: $256,300  (3) Niraparib: $257,100  Patients without gBRCA mutations:  (1) Placebo: $1,100  (2) Olaparib: $99,600  (3) Niraparib: $117,100 | PF-LYS:  All patients:  (1) Placebo: 0.34  (2) Olaparib: 0.76  (3) Niraparib: 0.92  Patients with gBRCA mutations:  (1) Placebo: 0.46  (2) Olaparib: 1.75  (3) Niraparib: 1.59  Patients without gBRCA mutations:  (1) Placebo: 0.32  (2) Olaparib: 0.62  (3) Niraparib: 0.78 | $100,000/ PF-YLS | All patients:  (1) Olaparib: $287,000/PF-LYS  (2) Niraparib: $235,000/PF-LYS  Patients with gBRCA mutations:  (1) Olaparib: $197,000/PF-LYS  (2) Niraparib: $226,000/PF-LYS  Patients without gBRCA mutations:  (1) Olaparib: $328,000/PF-LYS  (2) Niraparib: $253,000/PF-LYS | Maintenance niraparib and olaparib may not be cost-effective from a US health care sector perspective. |
| Dottino, J. A. et al (2019) ^(19)^ | Observation | (1) gBRCA testing and selective treatment of carriers (gBRCA only)  (2) gBRCA testing and HRD testing and selective treatment of either BRCA carriers or those with tumor HRD (gBRCA and HRD only)  (3) Treat all with niraparib | (1) Observation: $827  (2) gBRCA only: $46,157  (3) gBRCA and HRD only: $109,368  (4) Treat all: $169,127 | PF-QALYs:  (1) Observation: 0.29  (2) gBRCA testing or selective treatment: 0.48  (3) gBRCA testing plus HRD testing or selective treatment: 0.71  (4) Treat all: 0.74 | $100,000/PF-QALY | (1) gBRCA only: $243,092/PF-QALY  (2) gBRCA or HRD only: $269,883/PF-QALY  (3) Treat all: $2,200,000/PF-QALY | Maintenance niraparib is not cost effective compared with observation from US societal perspective. Treatment of patients with BRCA mutation alone or with HRD+ patients are preferred strategies compared to a treat all strategy. |
| Guy, H. et al (2019) ^(20)^ | RS | (1) Niraparib  (2) Olaparib  (3) Rucaparib | gBRCA mutation:  (1) RS: $95,628  (2) Niraparib: $396,802  (3) Olaparib: $405,601  (4) Rucaparib：$595,510  non-gBRCA mutation:  (1) RS: $100,724  (2) Niraparib: $333,322  (3) Olaparib: $355,558  (4) Rucaparib: $406,883 | QALYs:  gBRCA mutation:  (1) RS: 2.801  (2) Niraparib: 7.212  (3) Olaparib: 6.532  (4) Rucaparib: 6.050  non-gBRCA mutation:  (1) RS: 2.231  (2) Niraparib: 4.379  (3) Olaparib: 2.756  (4) Rucaparib: 3.948  LYGs:  gBRCA mutation:  (1) RS: 3.564  (2) Niraparib: 8.824  (3) Olaparib: 8.824  (4) Rucaparib: 7.437  non-gBRCA mutation:  (1) RS: 2.816  (2) Niraparib: 5.351  (3) Olaparib: 3.727  (4) Rucaparib: 4.868 | $150,000/QALY | ICER versus RS  gBRCA mutation:  (1) Niraparib: $68,287/QALY (2) Olaparib: $83,078/QALY  (3) Rucaparib: $153,866/QALY  non-gBRCA mutation:  (1) Niraparib: $108,287/QALY (2) Olaparib: $485,304/QALY (3) Rucaparib: $178,382/QALY | Niraparib was cost-effective compared to RS, and dominated olaparib and rucaparib with decreased costs and increased QALYs from a US payer perspective. |
| Cheng, L. J. et al  (2021) ^(31)^ | Observation | (1) All-olaparib  (2) Olaparib with BRCA mutation | (1) All-olaparib: $152,868  (2) Olaparib with BRCA mutation: $100,323  (3) Observation: $85,989 | QALYs：  (1) All-olaparib: 2.7447  (2) Olaparib with BRCA mutation: 2.2457  (3) Observation: 2.0820 | $34,047/QALY | All-olaparib vs. Observation:  $100,926 /QALY  Olaparib with BRCA mutation vs. Observation:  $87,566 /QALY  All-olaparib vs. Olaparib with BRCA mutation:  $105,308/QALY | Olaparib is not cost-effective when used with or without restriction by BRCA1/2 mutation status from the perspective of the Singapore healthcare system. |
| Leung, J. H. et al (2021) ^(35)^ | Placebo | (1) Olaparib  (2) Niraparib | All:  (1) Placebo: NT$157,693  (2) Olaparib: NT$992,226  (3) Niraparib: NT$1,604,913  Patients with gBRCA mutation:  (1) Placebo: NT$157,693  (2) Olaparib: NT$992,226  (3) Niraparib: NT$1,604,913  Patients with non-gBRCA mutation:  (1) Placebo: NT$157,693  (2) Olaparib: NT$992,226  (3) Niraparib: NT$1,604,913 | PF-LYs:  Platinum-sensitive recurrent ovarian cancer patients:  (1) Placebo: 0.35  (2) Olaparib: 0.81  (3) Niraparib: 0.97  Patients with gBRCA mutation:  (1) Placebo: 0.46  (2) Olaparib: 1.59  (3) Niraparib: 1.75  Patients with non-gBRCA mutation:  (1) Placebo: 0.33  (2) Olaparib: 0.62  (3) Niraparib: 0.78 | NT$2,602,404/PF-LY  ($93,478/PF-LY) | All:  (1) Olaparib: NT$1,804,785/PF-LY  (2) Niraparib: NT$2,340,265/PF-LY  Patients with gBRCA mutation:  (1) Olaparib: NT$737,219.5/PF-LY  (2) Niraparib: NT$1,120,139/PF-LY  Patients with non-gBRCA mutation:  (1) Olaparib: NT$2,828,924/PF-LY  (2) Niraparib: NT$3,216,044/PF-LY | Both olaparib and niraparib were cost-effective compared to placebo, and olaparib was more cost-effective than niraparib, from a single-payer perspective in Taiwan (China).  Olaparib or niraparib maintenance in patients with gBRCA mutations is more cost-effective than in patients without gBRCA mutations. |
| Shu, Y. et al (2022) ^(39)^ | Placebo | Olaparib | (1) Placebo: $13,022.68  (2) Olaparib: $56,315.60 | QALYs:  (1) Placebo: 2.86  (2) Olaparib: 3.42 | $31,498.70/QALY | $77,620.56/QALY | Olaparib as maintenance therapy is not considered to be cost effective, compared with placebo for patients with BRCA mutations from the perspective of the Chinese healthcare system. |
| **Maintenance therapy after first-line platinum-based chemotherapy for newly diagnosed ovarian cancer** | | | | | | | |
| Armeni, P. et al  (2020) ^(22)^ | AS | Olaparib | (1) AS: €97,043  (2) Olaparib: €124,359 | QALYs:  (1) AS: 4.88  (2) Olaparib: 7.29  LYs:  (1) AS: 6.21  (2) Olaparib: 9.08 | €16,372/QALY | €9,515/LY  €11,345/QALY | Olaparib maintenance therapy was cost-effective for patients with gBRCA mutation from the Italian NHS perspective. |
| Barrington, D. A. et al (2020) ^(23)^ | Observation | Niraparib | overall group:  (1) Observation: $5.8 billion (2) Niraparib: $20.5 billion  HRD group:  (1) Observation: $3.0 billion (2) Niraparib: $14.8 billion  BRCA mutation:  (1) Observation: $1.6 billion (2) Niraparib: $8.2 billion  HRD without BRCA mutation:  (1) Observation: $1.3 billion (2) Niraparib: $6.1 billion  non-HRD group:  (1) Observation: $2.8 billion (2) Niraparib: $7.1 billion | OS (months):  overall group:  (1) Observation: 24.6  (2) Niraparib: 41.4  HRD group:  (1) Observation: 31.2  (2) Niraparib: 65.7  BRCA mutation:  (1) Observation: 32.7  (2) Niraparib: 66.3  HRD without BRCA mutation:  (1) Observation: 24.6  (2) Niraparib: 58.8  non-HRD group:  (1) Observation: 16.2  (2) Niraparib: 24.3 | $100,000/QALY | overall group:  $72,829/QALY  HRD group:  $56,329/QALY  BRCA mutation:  $58,348/QALY  HRD without BRCA:  $50,914/QALY  non-HRD group:  $88,741/QALY | Niraparib maintenance therapy was cost effective from the US third-party payer perspective. Cost effectiveness was improved when analyzing those patients with HRD and BRCA mutations. |
| Gonzalez, R. et al  (2020) ^(24)^ | Biomarker-directed PARPi | PARPi-for-all | Veliparib:  (1) Biomarker-directed: $167,334  (2) For all: $286,715  Niraparib:  (1) Biomarker-directed: $98,188  (2) For all: $166,269  Olaparib plus Bevacizumab:  (1) Biomarker-directed: $260,671  (2) For all: $366,506 | Not report | $150,000/QA-PFY | PARPi-for-all vs biomarker-directed  Veliparib:  $1,513,495/QA-PFY  Niraparib:  $593,250/QA-PFY  Olaparib plus Bevacizumab:  $3,347,915/QA-PFY | The PARPi-for-all maintenance strategy was not cost-effective when compared to a biomarker-directed approach from the US third-party payer. |
| Muston, D. et al  (2020) ^(25)^ | Surveillance | Olaparib | (1) Surveillance: $350,735  (2) Olaparib: $503,280 | QALYs:  (1) Surveillance: 5.29  (2) Olaparib: 8.22  LYs:  (1) Surveillance: 6.52  (2) Olaparib: 10.15 | $100,000/QALY | $51,986/QALY  $42,032/LY | First-line maintenance olaparib was cost-effective compared with surveillance for patients with gBRCA mutation from a US third-party payer perspective. |
| Penn, C. A. et al  (2020) ^(26)^ | Observation | Base case 1 (with a BRCA variant):  (1) Olaparib  (2) Olaparib/Bev  (3) Bev  (4) Niraparib  Base case 2 (with HRD without a BRCA variant):  (1) Olaparib/Bev  (2) Bev  (3) Niraparib  Base case 3 (with HRP):  (1) Olaparib/Bev  (2) Bev  (3) Niraparib | (1) Observation: $3051  (2) Olaparib: $418,848  (3) Olaparib/Bev: $545,758  (4) Bev: $133,591  (5) Niraparib: $492,226 | PF-LYS:  Base case 1:  (1) Olaparib:2.23  (2) Olaparib/Bev: 1.48  (3) Bev: 0.26  (4) Niraparib: 0.46  Base case 2:  (1) Olaparib/Bev: 0.86  (2) Bev: 0.18 (3) Niraparib: 0.46  Base case 3:  (1) Olaparib/Bev: 0.25  (2) Bev: 0.23 (3) Niraparib: 0.05 | $100,000/PF-LYS | Base case 1:  (1) Olaparib: $186,777/PF-LYS  (2) Olaparib/Bev: $366,199/PF-LYS  (3) Bev: $508,434/PF-LYS  (4) Niraparib: $1,069,627/PF-LYS  Base case 2:  (1) Olaparib/Bev: $629,347/PF-LYS  (2) Bev: $717,255/PF-LYS  (3) Niraparib: $1,072,754/PF-LYS  Base case 3:  (1) Olaparib/Bev: $2,153,600/PF-LYS  (2) Bev: $557,865/PF-LYS  (3) Niraparib: $10,870,576/PF-LYS | Maintenance olaparib and niraparib is not cost-effective, regardless of molecular signature from perspective of the US health care sector. |
| Wolford, J. E. et al (2020) ^(28)^ | (1) NPBC  (2) Bev + NPBC | (1) Niraparib  (2) Rucaparib  (3) Olaparib | (1) NPBC: $39,579  (2) Bev + NPBC: $85,309  (3) Niraparib: $132,790  (4) Rucaparib: $133,096  (5) Olaparib: $114,289 | QALmonths：  (1) NPBC: 5.8  (2) Bev+ NPBC: 6.8  (3) Niraparib: 7.0  (4) Rucaparib: 8.0  (5) Olaparib: 7.0 | Not set | (1) Niraparib：  vs NPBC: $74,569/QALmonth  vs Bev+NPBC: $189,924/QALmonth  (2) Rucaparib:  vs NPBC: $41,563/QALmonth  vs Bev+NPBC: $38,230/QALmonth  (3) Olaparib:  vs NPBC: $59,768/QALmonth  vs Bev+NPBC: $115,920/QALmonth | Given that no WTP threshold was set, this paper could not conclude which strategy was cost effective. |
| Tan, D. S. et al  (2021) ^(34)^ | RS | Olaparib | (1) RS: $201,63  (2) Olaparib: $242,823 | QALYs：  (1) RS: 5.68  (2) Olaparib: 8.53  LYs：  (1) RS: 7.82  (2) Olaparib: 11.26 | $43,799/QALY | $14,470/QALY  $11,973/LY | Olaparib maintenance was cost-effective versus RS for patients with BRCA1/2 mutation from a healthcare payer perspective of Singapore. |
| Moya-Alarcón, C. et al (2021) ^(37)^ | No maintenance | Olaparib | (1) No maintenance: €99,601.64  (2) Olaparib: €129.320.07 | QALYs:  (1) No maintenance: 3.57  (2) Olaparib: 5.56 | €25,000/QALY | €14,653.20/QALY | Olaparib maintenance treatment is cost-effective in advanced high-grade serous ovarian carcinoma patients with BRCA mutations from the perspective of the Spanish National Health Service. |
| Elsea, D. et al (2022) ^(38)^ | Bevacizumab | Olaparib plus bevacizumab | (1) Bevacizumab: $238,554  (2) Olaparib plus bevacizumab: $402,763 | QALYs:  (1) Bevacizumab: 4.72  (2) Olaparib plus bevacizumab: 7.61  LYs:  (1) Bevacizumab: 6.12  (2) Olaparib plus bevacizumab: 9.55 | $100,000/QALY | $56,863 /QALY  $47,910 /LY | Olaparib plus bevacizumab is cost effective compared with bevacizumab alone in HRD ovarian cancer patients from the perspective of a US healthcare system. |
| **Breast cancer first-line therapy** | | | | | | | |
| Saito, S. et al  (2019) ^(21)^ | Standard chemotherapy without BRCA1/2 mutation | Olaparib monotherapy with BRCA1/2 mutation | (1) Standard chemotherapy: JPY 1,816,955  (2) Olaparib monotherapy: JPY 2,353,107 | QALYs:  (1) Standard chemotherapy alone: 0.417  (2) Olaparib monotherapy with BRCA1/2 mutation profiling: 0.454  PF-LYs:  (1) Standard chemotherapy alone: 0.543  (2) Olaparib monotherapy with BRCA1/2 mutation profiling: 0.583 | $107,143/QALY | $131,047/QALY  $120,068/PF-LYS | BRCA1/2 mutation profiling to target olaparib use is not a cost-effective strategy compared with standard chemotherapy for metastatic breast cancer from the perspective of a Japanese healthcare payer. |
| Olry de Labry Lima, A et al (2021) ^(33)^ | Standard group Scenario 1: capecitabine after anthracyclines/taxanes  Scenario 2:  eribulin after anthracyclines/taxanes and capecitabine | Talazoparib | Scenario 1:  (1) Standard group: €26,683.90  (2) Talazoparib: €84,360.86  Scenario 2:  (1) Standard group: €33,195.36  (2) Talazoparib: €92,515.05 | QALYs：  (1) Standard group: 0.83  (2) Talazoparib: 1.09 | €21,000, €24,000, €25,000, €60,000 /QALY | ICUR：  Utility values from Paracha (2016) ^(53)^:  Scenario 1: €252,420/QALY  Scenario 2: €259,609/QALY  Utility values from Ettl (2018) ^(54)^:  Scenario 1: €223,518/QALY  Scenario 2: €229,884/QALY | Talazoparib is not cost-effective versus standard treatment from the perspective of the Spanish National Health System |
| **Pancreatic cancer maintenance therapy** | | | | | | | |
| Wu, B. et al  (2020) ^(29)^ | Placebo | Olaparib | Overall cost:  (1) Placebo: $10,141  (2) Olaparib: $138,407  Progression-free cost:  (1) Placebo: $2,665  (2) Olaparib: $134,952 | Overall LYs:  (1) Placebo: 2.094  (2) Olaparib: 2.673  Progression-free LYs:  (1) Placebo: 0.950  (2) Olaparib: 1.888  QALYs:  (1) Placebo: 1.380  (2) Olaparib: 1.863  Progression-free QALYs:  (1) Placebo: 0.726  (2) Olaparib: 1.417 | $200,000/QALY | $221,789/LY  $141,003/PF-LYS  $265,290/QALY  $191,596/PFS-QALY | In the base case analysis, olaparib was not cost-effective versus placebo from the US payer perspective. PSA showed a nearly 54% probability of maintenance olaparib being a cost-effective strategy at the threshold of $200,000/QALY. |
| Zhan, M. et al  (2020) ^(30)^ | Placebo | Olaparib | (1) Placebo: $30,857.83  (2) Olaparib: $54,402.18 | QALYs:  (1) Placebo: 1.22  (2) Olaparib: 1.91 | $28,255.55/QALY | $34,122.25/QALY | Compared with placebo, maintenance olaparib for metastatic pancreatic cancer patients with a gBRCA mutation is not cost-effective from the Chinese society’s perspective. |
| Li, N. et al  (2021) ^(32)^ | Placebo | Olaparib | China:  (1) Placebo: $2,773  (2) Olaparib: $61,477  US:  (1) Placebo: $91,623  (2) Olaparib: $208,504 | QALYs：  China:  (1) Placebo: 5.22  (2) Olaparib: 13.99  US:  (1) Placebo: 5.22  (2) Olaparib: 13.99 | China: $30,829/QALY  US: $50,000/QALY | China: $6,694/QALY  US: $13,327/QALY | Olaparib was more cost effective than placebo for the maintenance therapy of pancreatic cancer with a gBRCA mutation from the perspective of US and China healthcare systems. |
| **Prostate Cancer** | | | | | | | |
| Su, D. et al  (2020) ^(27)^ | Standard care  (Treated with  enzalutamide  or abiraterone) | Olaparib | Scenario A: patients with at least one of the BRCA1, BRCA2 and ATM gene alterations  (1) Standard care: $17,243  (2) Olaparib: $24,626    Scenario B: patients with at least one of the 15 prespecified gene alterations  (1) Standard care: $55,476  (2) Olaparib: $48,526 | QALYs:  Scenario A:  (1) Standard care: 0.109 (2) Olaparib: 0.173  Scenario B:  (1) Standard care: 0.313 (2) Olaparib: 0.380  LYs:  Scenario A:  (1) Standard care: 0.221 (2) Olaparib: 0.321  Scenario B:  (1) Standard care: 0.540 (2) Olaparib: 0.665 | $150,000/QALY | Scenario A: $116,903/QALY  Scenario B: Dominance | The genomic test-directed olaparib is a preferred option compared with standard care for men with mCRPC who had any of all 15 prespecified genes from the US payer perspective. |
| Li, Y. et al (2021) ^(36)^ | Control treatment (Treated with  enzalutamide  or abiraterone) | Olaparib | (1) Standard care: $80,154  (2) Olaparib: $157,732 | LYs:  (1) Standard care: 1.44  (2) Olaparib: 1.85  QALYs:  (1) Standard care: 0.95  (2) Olaparib: 1.26 | $200,000/QALY | $189,215/LY  $248,248/QALY | Olaparib is not cost effective in comparison with standard care in mCRPC patients with specific gene mutations from the US payer perspective. |
| Xu, C. et al (2022) ^(40)^ | Control treatment (Treated with  enzalutamide  or abiraterone) | Olaparib | China:  (1) Control: ¥193,337.81  (2) Olaparib: ¥287,011.03  US:  (1) Control: $310,607.38  (2) Olaparib: $240,932.18 | QALYs:  (1) Control: 0.73  (2) Olaparib: 0.96 | China: ¥217,341/QALY  US: $150,000/QALY | China: ¥392,727.87/QALY  US: dominant | Olaparib is not cost effective in treatment of patients with mCRPC in China, but it is cost saving in the US from the perspective of health service. |

WTP, willingness to pay; QALY, quality adjusted life-year; ICER, incremental cost-effectiveness ratio; PFS, progression-free survival; PF-YLS, progression-free year of life saved; gBRCA, germline BRCA; US, the United States; PF-QALY, progression-free quality adjusted life-year; RS, routine surveillance; AS, active surveillance; NHS, National Health Service; OS, Overall Survival; HRD, homologous recombination deficient; PARP, Poly ADP-ribose polymerase; HRP, homologous recombination proficiency; Bev, Bevacizumab; NPBC, Non-platinum based chemotherapy; QALmonth, quality of adjusted ovarian cancer life in months; LY, life-year; MCRPC, Metastatic Castration-Resistant Prostate Cancer.
